# O princípio da precaução nas ações preventivas: considerações sobre uma ausência

**DOI:** 10.1590/0102-311XPT195125

**Published:** 2026-10-05

**Authors:** Charles Dalcanale Tesser

**Affiliations:** 1 Centro de Ciências da Saúde, Universidade Federal de Santa Catarina, Florianópolis, Brasil.

**Keywords:** Princípio da Precaução, Medicina Preventiva, Iatrogenia, Prevenção Quaternária, Precautionary Principle, Preventive Medicine, Iatrogenic Disease, Quaternary Prevention, Principio de la Precaución, Medicina Preventiva, Iatrogenia, Prevención Cuaternaria

## Abstract

O princípio da precaução (PP) pode ser entendido como: em caso de grave dano ou catástrofe para o meio ambiente ou a saúde humana, em que há incerteza científica sobre suas causas e/ou incidência, devem ser tomadas medidas para evitar o dano. Todavia, o PP não tem sido usado para evitar danos iatrogênicos graves de ações derivadas de ações de prevenção primária (P1) ou secundária (P2) na medicina ou nos sistemas de saúde. Este ensaio, após sintetizar uma proposta para esse uso e defender sua adequação, apresenta quatro hipóteses compreensivas interligadas e sinérgicas sobre por que ele não tem ocorrido: (a) tal uso é muito anti-intutitivo devido à semelhança teleológica entre o PP e a prevenção e suas diferenças contextuais; (b) a expansão moral da medicina para o futuro vem gerando uma supremacia preventivista que amplia irrestritamente a tolerância à iatrogenia de P1/P2; (c) algumas dessas iatrogenias são anomalias (no sentido proposto por Thomas Kuhn) cuja identificação e mensuração são complexas, dificultando sua aceitação e valorização; (d) resultante das anteriores, há em curso uma naturalização já avançada da iatrogenia preventiva, que torna impensável a cogitação do PP para o seu manejo em casos graves duvidosos, deixando-o fora do horizonte da medicina preventiva e paradoxalmente tornando-o mais necessário.

## Abstract (en)

The precautionary principle (PP) consists of measures to be taken to avoid harm under serious damage or catastrophe to the environment or human health and scientific uncertainty about its causes and/or incidence. However, PP has not been used to prevent severe iatrogenic harm from actions derived from primary (P1) or secondary (P2) prevention actions in medicine or health systems. This essay synthesizes a proposal for this use, defends its adequacy, and offers four comprehensive, interconnected, and synergistic hypotheses as to why it is yet to occur: (a) such use is very anti-intuitive due to the teleological similarity between PP and prevention and their contextual differences; (b) the future moral expansion of medicine has generated a preventive supremacy that unrestrictedly expands tolerance to P1/P2 iatrogenesis; (c) some of these iatrogenic diseases are anomalies (in Thomas Kuhn’s sense) with complex identification and measurement, making it difficult to accept and value them; (d) the following ongoing naturalization of preventive iatrogenesis makes it unthinkable to consider PP for its management in serious cases of doubt, leaving it outside the horizon of preventive medicine and paradoxically making it more necessary.

## Abstract (es)

Se puede entender el principio de precaución (PP) como: en caso de daño grave o catástrofe para el medio ambiente o la salud humana, en el que exista incertidumbre científica sobre sus causas y/o incidencia, se deben adoptar medidas para evitar el daño. Sin embargo, el PP no se ha utilizado para evitar daños yatrogénicos graves de acciones derivadas de acciones de prevención primaria (P1) o secundaria (P2) en la medicina o en los sistemas de salud. Este ensayo, tras sintetizar una propuesta para este uso y defender su idoneidad, presenta cuatro hipótesis integrales, interconectadas y sinérgicas sobre por qué no se ha producido: (a) dicho uso es muy antiintuitivo, debido a la similitud teleológica entre el PP y la prevención y sus diferencias contextuales; (b) la expansión moral de la medicina hacia el futuro ha generado una supremacía preventiva que amplía sin restricciones la tolerancia a la yatrogenia de P1/P2; (c) algunas de estas yatrogenias son anomalías (en el sentido que propone Thomas Kuhn) cuya identificación y medición es compleja, lo que dificulta su aceptación y valoración; (d) como resultado de las anteriores, está en curso una naturalización ya avanzada de la yatrogenia preventiva, que hace impensable considerar el PP para su manejo en casos graves dudosos, dejándolo fuera del horizonte de la medicina preventiva y, paradójicamente, haciéndolo más necesario.

## Introdução

O princípio da precaução (PP) foi forjado na década de 1970, tendo sido considerado um princípio jurídico consuetudinário, uma regra de decisão ^1^ ^,^ ^2^ e também um princípio ético de nível médio ^3^, com variações e interpretações diversas. Embora haja uma polêmica sobre a melhor definição do PP ou mesmo se é possível uma definição unificada dele ^4^, uma estrutura aproximadamente comum das concepções do PP foi proposta ^5^ e ajuda a entender a maior parte das principais ideias envolvidas: dada uma situação de incerteza científica sobre as causas ou a incidência de um grave dano ao meio ambiente e/ou à saúde humana, não deixar que tal incerteza paralise órgãos governamentais, que devem ativamente tomar providências para evitar o dano ^6^ ^,^ ^7^.

Embora o PP seja aplicado na saúde pública, é raro ou inexistente o seu uso ou consideração na avaliação de ações preventivas médico-sanitárias com danos iatrogênicos graves, algumas recomendadas pelo Sistema Único de Saúde (SUS) e seus profissionais. Este ensaio objetiva, após sintetizar uma proposta para esse uso, apresentar hipóteses compreensivas sinérgicas e interligadas sobre por que o PP não tem sido aplicado nem cogitado nesse campo.

O próximo tópico resume uma abordagem orientadora da aplicação do PP à iatrogenia grave de ações preventivas e argumenta pela sua adequação. Os tópicos seguintes apresentam hipóteses compreensivas interligadas e sinérgicas sobre essa ausência. Dado o caráter hipotético das considerações apresentadas, optou-se pelo formato de ensaio, que permite uma livre apresentação de ideias e argumentos ^8^.

Como há grandes diferenças entre as ações de prevenção primária (P1 - evitar o aparecimento de uma doença) e secundária (P2 - antecipar a identificação de um adoecimento) ^9^ quanto aos seus objetivos, à produção, à disponibilidade e à interpretação das evidências sobre seus benefícios e danos e aos interesses respectivos envolvidos, é contraindicado generalizar ou igualar as diversas ações preventivas entre si nem estes dois níveis de prevenção. Entretanto, de um ponto de vista conceitual e ético, os argumentos favoráveis ou contrários a uma possível invocação do PP independem de a ação ser de P1 ou de P2. Essa é a razão estrita pela qual o grosso da argumentação que segue abrange P1 e P2 sempre que possível, dada a generalidade aqui visada. Vale destacar que, de um ponto de vista prático, clínico e/ou sanitário, somente se pode avaliar se é cabível ou não a aplicação do PP em uma específica ação de P1 ou de P2, frente aos saberes disponíveis sobre seus benefícios e danos.

## O lugar e a aplicação do princípio da precaução nas ações preventivas

O PP visa a evitar grandes danos ao ambiente ou à saúde humana. Já a prevenção de doenças e agravos objetiva evitar o adoecimento, o sofrimento e a mortalidade evitáveis, via P1 e P2. Devido a essa similaridade, o PP pode ser entendido como um modo de cuidado preventivo ^10^ assemelhado à P1 ^11^. Logo, há que evitar a confusão entre o PP e P1, e especificar em que sentido o primeiro será aqui tematizado: trata-se de usar o PP para evitar danos iatrogênicos graves de P1/P2.

Um exemplo esclarece esse uso: os critérios de liberação de sangue humano para transfusões são rígidos, já que ele pode veicular doenças graves. Por precaução, grande rigor foi usado na seleção do sangue para transfusões. Embora elas salvem vidas, não se tolera a produção de danos graves pelo sangue transfundido, e por isto foi usado o PP ^12^ para aumentar o rigor da seleção do sangue. Todavia, trataremos aqui apenas do seu uso em iatrogenias graves de P1/P2.

Alguns artigos ^13^ ^,^ ^14^ ^,^ ^15^ ^,^ ^16^ ^,^ ^17^ ^,^ ^18^ propuseram e fundamentaram uma orientação para o uso do PP em P1/P2, a seguir sintetizada, dentro de um contexto de redução de danos e medicalização desnecessária, chamado na atenção primária à saúde (APS) de prevenção quaternária (P4) ^19^. A P4 pode ser útil para melhorar a distribuição das ações preventivas, realocando parte de P1/P2 para a esfera das políticas públicas e da saúde pública - mais potentes para abordar determinações sociais, otimizar benefícios e minimizar danos de ações preventivas (por exemplo: redução de iniquidades e grandes desigualdades socioeconômicas, programas de vacinação [P1], programas organizados de rastreamento [P2]). Isso reduz o volume de tarefas preventivas dos profissionais da APS e libera mais o seu tempo para o cuidado clínico aos sensivelmente adoecidos ^19^. A P4 deve permear ao máximo tanto P1/P2 como a atenção aos já doentes.

Uma categorização de Geoffrey Rose ^20^ das ações preventivas em dois tipos permite um relevante divisor de águas, ao distinguir as de maior potencial iatrogênico. O primeiro tipo engloba ações que reduzem riscos de adoecimento sem intervenções artificiais, chamadas “redutivas”: redução do sedentarismo, do tabagismo, do alcoolismo, redução/eliminação dos alimentos multiprocessados e dos agrotóxicos etc. Tais medidas reduzem as artificialidades patogênicas presentes nos modos de viver, trabalhar, se alimentar, se divertir e se deslocar produzidas pelas sociedades urbanas industriais. Na prevenção redutiva, há forte consenso sobre várias ações pouco problemáticas, dado seu nulo ou muito baixo potencial de iatrogenia e o consenso científico sobre seus benefícios.

Já as intervenções preventivas “aditivas” envolvem a adição de “*um fator artificial, protetor e preventivo não existente na economia-fisiologia-ecologia das pessoas: vacinas, fármacos preventivos (hipotensores, hipolipemiantes), complementos alimentares artificiais (ou naturais em doses artificiais), rastreamentos etc.*” ^16^ (p. 5). Tais ações são fontes mais prováveis de iatrogenia grave, motivo pelo qual, quando são inicialmente propostas, devem ser exigidas evidências científicas de alta qualidade e convergentes sobre os seus resultados, quanto aos benefícios que interessam às pessoas ^21^ e aos seus danos iatrogênicos ^20^. Quando já estão em uso, essa avaliação deve envolver estudos observacionais de alta qualidade que mostram os resultados da ação no mundo real - o seu objetivo final. Uma atitude basal de resistência à sua introdução ou manutenção é necessária e justificada em P1/P2, devido à consensual maior valorização da não maleficência nas situações preventivas ^14^ ^,^ ^16^; resistência a ser superada pelas evidências empíricas mostrando grandes benefícios e poucos/leves danos.

Na avaliação dos benefícios e danos das ações preventivas aditivas não raramente há dúvidas e/ou polêmicas científicas. Quando essas dúvidas/polêmicas persistem e envolvem danos graves, isto caracteriza as condições clássicas e consensuais de aplicação do PP, visando a evitar tais danos. Aplicá-lo em ações de P1 ou P2 com iatrogenias graves é relativamente fácil: se for o caso de uma nova ação de P1/P2, uma forma simples, rápida, barata e sem consequências adversas de aplicar o PP é não recomendar/autorizar o seu uso. Se a ação já é usada, suspender ou inverter a sua recomendação positiva é necessário, simples (técnica e operacionalmente) e crucial para desimplementá-la, o que pode não ser rápido (por exemplo, em rastreamentos ^22^).

## Contextos díspares, similaridade de finalidades e atores

Uma primeira dificuldade para a aplicação do PP à P1/P2 é que o seu uso nas ações de prevenção é anti-intutitivo porque: (a) há grande distância temática e moral entre as ações de P1/P2 e as demais ações contra as quais, na sua grande maioria, o PP foi invocado e aplicado; (b) há grande semelhança moral/ética entre os objetivos de P1/P2 e do PP; e (c) há semelhança ou identificação dos defensores do PP com os operadores das ações de P1/P2.

(a) Uma enciclopédia de direito internacional assim descreve o contexto de aplicação do PP: “*os campos tipicamente afetados pelo princípio da precaução são a possibilidade de poluição persistente ou aguda, extinção de espécies, introdução de produtos novos e potencialmente nocivos no meio ambiente, ameaça à biodiversidade (organismos geneticamente modificados; diversidade biológica, proteção internacional), ameaças à saúde pública devido a novas doenças e técnicas, segurança alimentar (por exemplo, doença de Creutzfeldt-Jakob e preocupações com alimentos geneticamente modificados), física de altas energias e possivelmente experimentos catastróficos e outras novas questões de biossegurança (por exemplo, vida artificial, novas moléculas)*” ^23^ (tradução nossa).

Como se vê, os fins a que se destinam as ações/produtos afetados pela aplicação do PP, e estas próprias ações/produtos, são díspares, na grande maioria, em temática e contexto, do grosso das ações de P1/P2. Devido a essas grandes diferenças, não é intuitivo transpor a aplicação do PP para o contexto de P1/P2.

(b) Esse caráter contraintuitivo é intensificado por uma semelhança forte de finalidade entre as ações de P1/P2 e o PP, diferentemente da maior parte das ações e produtos aos quais o PP tem sido aplicado. Enquanto o PP visa a proteger os humanos e o ambiente de danos graves e/ou catastróficos, P1/P2 visam a proteger de doenças, sofrimentos e mortes evitáveis. A similaridade temporal, moral e ética salta aos olhos; o que os torna aparentados simbólica, cultural, linguística, afetiva e moralmente. Desse modo, é difícil distanciá-los e distingui-los, dificultando cogitar a aplicação do PP às ações de P1/P2 quando cabível.

(c) Por fim, outra semelhança entre P1/P2 e o PP é que, frequentemente, defensores ou operadores da aplicação do PP pertencem às mesmas (ou semelhantes) instituições/agências governamentais que recomendam ações de P1/P2, ou são os mesmos atores sociais. Ou seja, atores e/ou instituições invocadoras do PP são comumente também produtores ou partes de instituições maiores produtoras de recomendações de P1/P2. Ambos estão do “lado bom” e, como todos os “heróis”, temos dificuldades de encará-los, simultaneamente, como possíveis “vilões” ou agentes de ações recrimináveis.

Não se costuma partir do pressuposto de que tais atores sociais usarão comumente dois pesos e duas medidas nas suas avaliações e recomendações. Sendo dedicados a causas preventivas nobres, parece pouco crível que tenham dificuldade de ser rigorosos com as ações de P1/P2 ou que atenuem rigores protetivos e desviem de valores éticos consensuais por nobres intenções e finalidades de P1/P2, mas cujos resultados podem se mostrar gravemente danosos.

Essa “semelhança moral” nos fins e a proximidade ou identificação socioinstitucional dos atores envolvidos aumentam a dificuldade dos mesmos assumirem para as suas propostas de P1/P2 o rigoroso escrutínio do PP quando cabível, e dificultam para os movimentos sociais, profissionais de saúde, cientistas, usuários e agências reguladoras a adoção de um espírito crítico para as ações de P1/P2 gravemente iatrogênicas.

## A expansão moral da medicina e a supremacia preventivista

Vivemos em meio à expansão da jurisdição da medicina, a chamada medicalização social: uso de abordagens biomédicas em situações nas quais isto não era feito antes ^24^. Esse processo gera benefícios e danos, como sobrediagnósticos (diagnose de doenças que não apareceriam clinicamente ^25^), sobretratamentos (tratamentos de pessoas sobrediagnosticadas), proliferação de cuidados clínicos de baixo valor, aversão exagerada ao risco, custos cada vez maiores do cuidado médico etc.

Hofmann ^26^ destacou um aspecto desse processo que é a expansão moral da medicina - expansão das expectativas e aspirações de “fazer mais bem” - em três direções: (1) expansão dos cuidados e da doença para além da dor e do sofrimento; (2) expansão do cuidado do presente para o futuro, convergindo com a “medicina de vigilância” ^27^, que enfatiza a prevenção; e (3) redirecionamento da redução do bem-estar negativo à promoção do bem-estar positivo.

Parece crescer na biomedicina e na saúde pública um moralismo preventivista forte, disseminado e pouco questionado, que vem ampliando a tolerância aos danos progressiva e quase ilimitadamente, de modo a quase prescindir de comparações entre benefícios e danos ou os fazer apenas formalmente. A expansão da medicina para o futuro está gerando um dogmatismo moral pró-prevenção progressivo que se acopla às limitações epistêmicas e tecnológicas dos tratamentos das doenças crônicas no presente, pressionando silenciosa e fortemente os pesos e contrapesos éticos e as expectativas e valores sociais e culturais: o futuro não só vai se tornando semelhante em valor ao presente, mas talvez mesmo mais importante.

A valorização do futuro não é uma característica isolada da biomedicina. “*A modernidade é uma cultura do risco.* (...) *o futuro é continuamente trazido para o presente* (...)*. É como se um território* [o futuro] *fosse escavado e colonizado* (...) *mas essa colonização, por sua própria natureza, não pode se completar*” ^28^ (p. 11). A modernidade introduziu riscos novos que podem ter alto impacto na vida das pessoas e coletividades, e tenta controlá-los a partir da proliferação de sistemas especialistas abstratos (e de securitização, nítida tendência capitalista), demandando uma grande confiança nestes sistemas, a que retornaremos. Além de benefícios, a ciência e a tecnologia modernas geram riscos que sujeitam um número cada vez maior de pessoas, devendo ser, por isto, sujeitas à avaliação e discussão política, visando a suprimir as causas destes riscos ^29^. Beck ^29^ e Giddens ^28^ apontaram aspectos intrínsecos e problemáticos da presença e do manejo dos riscos nas sociedades modernas em um contexto amplo, em que as ações preventivas clínico-sanitárias são um exemplo não tematizado em profundidade, a não ser em linhas gerais, quanto à contingência e à mutabilidade dos saberes mediados por cada vez mais variados tipos de especialistas.

A hipervalorização atual das ações preventivas anda junto com a grande tolerância geral às iatrogenias da biomedicina, ambas se retroalimentando mutuamente. As duas são alimentadas e sustentadas pelo poder dos Estados, legitimadores da biomedicina, da ciência médica e da biotecnologia. Com esse triplo aval, a excessiva tolerância aos danos se legitimou, disseminou e progrediu, em meio à crença moderna generalizada e ingênua de que o avanço tecnológico é *a priori* benéfico, e de que se eventuais iatrogenias preventivas são toleradas pelas agências reguladoras e pelos médicos, certamente são compensadas por benefícios significativos e valiosos, com o desenvolvimento científico a atenuá-las ou eliminá-las no futuro.

A proeminência social, cultural e sanitária das doenças crônicas associou-se com a medicalização social e a progressiva influência da indústria farmacêutica na produção do saber e das práticas preventivas ^30^, e esta conjunção alimenta o aumento progressivo e irrefletido de tolerância (moral e social, culturalmente disseminada) aos danos iatrogênicos das novas e das já usadas ações preventivas. Aliás, a priorização da prevenção é defendida por alguns como “a” direção promissora para o desenvolvimento epistêmico e tecnológico biomédico (vide a medicina 4P: preditiva, preventiva, personalizada e participativa ^31^).

Outra força ampliadora dessa tolerância excessiva é a extensão da maior (e já grande) tolerância à iatrogenia curativa para a esfera das ações preventivas via incorporação de situações de alto risco aos diagnósticos de patologias e síndromes ^13^, em que a iatrogenia das ações preventivas passa a ser manejada do mesmo modo que a do cuidado aos sensivelmente adoecidos. Com isso, as suas especificidades éticas e técnicas são diluídas e apagadas nas águas turvas do oceano altamente tolerado dos comuns efeitos adversos das intervenções diagnósticas e terapêuticas “curativas”. Isso faz com que os critérios de legitimação epistêmica de ações de P1/P2 sejam desacoplados do rigor da exigência de evidências empíricas de alto padrão e hierarquia convergentes, que mostrem um grande benefício líquido no balanço benefícios/danos, com poucos danos para a sua aprovação/manutenção.

O rigor avaliativo da segurança/eficácia vai sendo relativizado pela intocável e inquestionável prioridade de ampliar e fazer avançar ações de P1/P2, devido à nobreza moral e à necessidade quase compulsiva de mais prevenção; que funcionam juntas como uma nova e subliminar força cultural, social e científica pouco tematizada criticamente, em sinergia com a tolerância excessiva aos danos.

Hofmann ^26^ propôs duas estratégias para o manejo da citada tripla expansão moral da biomedicina, para aproveitar seus benefícios e reduzir seus danos, via a exploração de duas assimetrias: a primeira é epistêmica e referente à incerteza sobre o presente e o futuro. A incerteza na medicina (e seu erro prognóstico) é menor no presente do que no futuro. Essa assimetria indica priorizar/direcionar o desenvolvimento epistêmico e tecnológico mais para o cuidado dos sofrimentos e doenças presentes, reduzindo a incerteza e o erro prognóstico, o que tenderia a reduzir danos iatrogênicos preventivos.

Outra diferença entre o cuidado ao adoecimento presente e o voltado ao futuro reforça essa primeira assimetria: as ações preventivas devem poder garantir um benefício líquido como seu resultado, diferentemente da situação clínica em que a pessoa doente solicita cuidado. Como disse Sackett ^32^, as situações de procura pelo doente e a oferta de ações preventivas (P1/P2) são: “*diferentes em suas obrigações e promessas implícitas aos indivíduos cujas vidas modificam. Quando os pacientes me procuraram em busca de ajuda para suas doenças sintomáticas estabelecidas, prometi apenas fazer o meu melhor e nunca garanti que minhas intervenções os melhorariam. Embora muitas das minhas intervenções tenham sido validadas em ensaios clínicos randomizados, a necessidade de intervir* (...) *me forçou a usar tratamentos justificados apenas com base em experiência anterior, opiniões de especialistas e princípios da fisiologia e farmacologia. Mas certamente a promessa fundamental que fazemos quando solicitamos ativamente aos indivíduos e os exortamos a aceitar intervenções preventivas deve ser a de que, em média, eles se sairão melhor com isso. Consequentemente, a presunção que justifica a assertividade agressiva com que perseguimos os saudáveis desavisados deve ser baseada no mais alto nível de evidência randomizada de que nossa manobra preventiva, de fato, fará mais bem do que mal. Sem evidências de ensaios clínicos randomizados positivos (e, melhor ainda, revisões sistemáticas de ensaios clínicos randomizados), não podemos justificar a solicitação aos saudáveis para que aceitem qualquer intervenção preventiva*” ^32^ (p. 363, tradução nossa).

Essa diferença deriva da grande e maior valorização já indicada anteriormente da não maleficência em P1/P2, unânime na literatura ^32^ ^,^ ^33^ ^,^ ^34^ ^,^ ^35^ ^,^ ^36^ ^,^ ^37^, mas que parece estar sendo cada vez menos praticada.

A segunda estratégia proposta por Hofmann ^26^ vem da assimetria ética entre noções negativas, como mal, doença, dor, sofrimento e morte; e noções positivas, como bem, saúde, prazer, felicidade e vida. As primeiras são, para o setor saúde (sistemas de saúde e seus profissionais) mais fundamentais, bem definidas e operacionalmente importantes do que as segundas. Essa assimetria ética direciona a expansão da biomedicina para fenômenos associados às noções negativas e no presente. Tais estratégias vão em direção inversa à da expansão moral da medicina, porém são subvalorizadas frente à cultura e à moral preventivistas, o que contribui para a ausência do PP.

Ainda outra questão moral afasta implicitamente o PP: o fato bem documentado de que as pessoas em situações ou temas sensíveis como a prevenção de doenças graves agem/decidem priorizando valores morais, com base no que consideram “certo”, mesmo que a altos custos ou com resultados prejudiciais. Há uma troca de poucos benefícios valiosos associadas ao “certo a se fazer”, apresentados com palavras salvacionistas (“salvar vidas” ou “evitar mortes”) pela aceitação de muitos danos supostamente ou apresentados como menores ^38^. Isso dificulta uma postura utilitarista e racional das ações preventivas e empurra a decisão individual e a opinião pública para um quase fundamentalismo preventivista moralista, no qual a prevenção é o “certo”. Por outro lado, gera um relativismo moral unilateral subvalorizador da proteção contra danos iatrogênicos, contribuindo para o PP continuar afastado. Cria-se uma obrigação moral de prevenção ^39^ ^,^ ^40^.

Assim, pode-se cogitar que desde o final do século XX intensificou-se uma colonização ideológica preventivista do imaginário social e dos profissionais de saúde, geradora de uma supremacia preventivista, de modo que o espírito crítico quanto à P1/P2 foi e continua sendo inibido, enfraquecendo/bloqueando na prática a consensual grande valorização discursiva da não maleficência em P1/P2.

No caso dos rastreamentos de alguns cânceres, gera-se também uma “vulnerabilidade patogênica” ^41^ aos sobrediagnósticos, definida como a geração de efeitos adversos produzidos por ações visando a reduzir vulnerabilidades, que aumentam as já existentes ou geram novas vulnerabilidades, com limitação ou prejuízo da agência das pessoas. Esses rastreamentos visam a reduzir a vulnerabilidade aos cânceres, mas criam a vulnerabilidade patogênica aos sobrediagnósticos, difíceis de entender (complexos), polêmicos cientificamente e inacessíveis à percepção das pessoas.

## O caráter complexo e anômalo de algumas iatrogenias da prevenção

Algumas iatrogenias são epistemicamente complexas e suscitam dúvidas e/ou polêmicas científicas. Dois danos iatrogênicos gravíssimos são o sobrediagnóstico e o sobretratamento, não raros em rastreamentos (P2) e cada vez mais importantes, devido ao aumento progressivo da sensibilidade dos exames bioquímicos e imagéticos diagnosticadores de doenças e fatores de riscos ^42^. Os rastreamentos são complexos e de avaliação difícil e, no caso dos cânceres (neoplasias malignas), são completamente inacessíveis à percepção e à experiência dos profissionais e dos pacientes, podendo ser entendidos aproximadamente como uma “anomalia”, conforme Thomas Kuhn ^43^.

A teoria de Kuhn ^43^ trata do desenvolvimento dos saberes científicos. Para o autor, em uma disciplina ou área de uma ciência madura, os pesquisadores trabalham sob a vigência de um paradigma: o conjunto de realizações, saberes e métodos desenvolvidos e consensualmente aceitos pela sua comunidade científica, que orientam a então chamada “ciência normal”. Nela, as teorias, os modos aprendidos por semelhança de resolução de problemas e os métodos já consagrados (no paradigma) permitem vislumbrar uma imagem geral do domínio da realidade investigado, como se fosse a foto orientadora da montagem de um quebra-cabeça. Uma anomalia ocorre quando os resultados observados contradizem persistentemente as expectativas previstas pelo paradigma; não fazem sentido no conjunto de seus saberes, não se encaixam no que é razoável, plausível, esperado no quadro paradigmático.

As neoplasias malignas (cânceres), embora na sua grande maioria não se conheça suas causas, são tidas, em geral, como progressivas e mortais - ainda que alguns cânceres sejam conhecidos como muito lentos na sua progressão, esta seja geralmente local, sem ou com pouca produção de metástases a distância. Um pioneiro modelo, muito usado até hoje, é o modelo da história natural das doenças, de Leavell & Clark ^9^, que, no caso de vários cânceres, poderia ser simplificadamente resumido à afirmação: se não identificadas e tratadas precocemente, essas neoplasias malignas tendem a se desenvolver, invadir os gânglios linfáticos adjacentes e se espalhar pelo corpo em metástases ^44^, ameaçando a vida. Para esses cânceres não está previsto que não evoluam progressivamente.

O conteúdo do modelo é específico para cada tipo de câncer, e é fornecido pelos saberes médico-epidemiológicos correntes, sendo modificado conforme novos saberes são aceitos pela comunidade de médicos e pesquisadores. Porém, devido à dinâmica da construção, legitimação e socialização do saber científico, conforme Fleck ^45^, o modelo tende a ser recheado com saberes já consagrados, que nem sempre correspondem aos mais avançados dos círculos superespecializados de pesquisadores (chamados pelo autor “círculos esotéricos”), que podem demorar para ser legitimados.

Assim, os conteúdos dos modelos orientadores das atividades clínicas e sanitárias são os já consagrados, um tanto simplificados, dogmatizados ^45^, sistematizados e harmonizados com o paradigma, no linguajar de Kuhn. Em relação ao consagrado no caso de alguns cânceres malignos, há saberes e padrões-ouro consensuados, estáveis e seguidos internacionalmente para a classificação de seus estágios de progressão desde a metade do século XX ^46^. Isso faz com que seja amplamente aceita a afirmação genérica de que quanto mais cedo são diagnosticados, melhor a chance de cura, realizada à base de tratamentos agressivos e muito iatrogênicos: ressecção cirúrgica da neoplasia, quimioterapia e/ou radioterapia. Por isso, é relativamente consensual que quanto mais tardio o diagnóstico, pior o prognóstico, e que, como regra geral, é melhor prevenir do que remediar.

Ao longo da segunda metade do século XX, foram desenvolvidas técnicas de detecção precoce de alguns desses cânceres aplicáveis em pessoas assintomáticas (rastreamentos) ^47^. Entretanto, altas proporções de sobrediagnósticos de cânceres de alta relevância sanitária, como os de mama, tireoide, próstata e pele, são uma realidade aceita ^48^ e problemática para a saúde pública ^49^ e a justiça social ^50^. Ou seja, esses cânceres, classificados como malignos histopatologicamente, podem não avançar. Isso é contraintuitivo ^51^ e teoricamente anômalo, em certa medida, a ponto de terem sido propostas mudanças de nomenclatura para os cânceres diagnosticados por rastreamentos (a maior fonte de sobrediagnósticos), para que não fossem designados “câncer” ^52^, o que não foi efetivado, salvo em pouquíssimos outros casos.

Assim, os sobrediagnósticos podem ser aproximadamente considerados anomalias desafiadoras dos saberes oncológicos amplamente aceitos para alguns tipos de câncer. Ainda que em certas áreas altamente especializadas de pesquisa o sobrediagnóstico tenha sido incorporado, e que em poucas situações clínicas hoje se pratique uma “vigilância ativa” em casos de baixa agressividade (em cânceres de próstata e tireoide, por exemplo), os sobrediagnósticos em geral não parecem ser valorizados como os danos catastróficos que são na avaliação do balanço benefícios/danos. Doutra parte, nesses casos, tal conduta expectante de vigilância, por sua vez, gera nova ordem de iatrogenias nessas pessoas, que não sofrem do sobretratamento, mas passam, via rastreamento, da condição de saudáveis para a de cancerosos menos graves com uma espada de Dâmocles sobre a cabeça permanentemente, sobretudo quando dos exames de imagem e biópsias de monitoramento. O caráter anômalo dos sobrediagnósticos no grosso da cultura geral e médica provavelmente fomenta fortes dúvidas explícitas ou implícitas sobre sua existência, prevalência e relevância; o que parece contribuir fortemente para manter afastado e impensável o PP nesses rastreamentos.

Outra propriedade complexa que dificulta a aceitação plena do sobrediagnóstico na comunidade médica e entre os leigos é seu caráter de ser imperceptível aos profissionais e pacientes. Carter ^53^ chama a essa característica de “inacessibilidade epistêmica”: cada caso sobrediagnosticado e sobretratado é totalmente imperceptível, já que o sobrediagnóstico pode apenas ser percebido indireta, retrospectiva e anonimamente em estudos epidemiológicos, por meio de estatísticas incapazes de identificar pessoalmente as vítimas ^26^. Todas as pessoas sobrediagnosticadas se sentem salvas pelo rastreamento e pelo tratamento (sobretratamento), quando foram altamente prejudicadas, gerando o “paradoxo da popularidade”: quanto mais rastreamentos houver e mais sobrediagnósticos forem produzidos, mais populares se tornam os rastreamentos, pois as pessoas por eles prejudicadas se somam na defesa dos seus supostos benefícios por se sentirem salvas. A catástrofe iatrogênica dos sobrediagnósticos não só é imperceptível, fazendo com que ninguém possa reclamar ou demandar reparos/indenizações pelos danos, ela também é autorretroalimentadora via paradoxo da popularidade ^54^.

Os sobrediagnósticos são considerados dos mais importantes danos iatrogênicos dos rastreamentos, sobretudo nos cânceres. Isso é compreensível pelo seu caráter catastrófico, que se abate sobre pessoas sadias rastreadas, as quais sentem suas vidas ameaçadas, não padecem do câncer em si, mas padecem do sobrediagnóstico e do sobretratamento (sem saberem que se trata de sobrediagnóstico), pois creem, como seus médicos, que estão com sua vida ameaçada pelo câncer, e ficam por anos realizando *follow-ups* de um diagnóstico de câncer que pode sempre retornar, a lhes alterar a identidade para toda a vida ^55^. Nesse sentido, o dano do sobrediagnóstico não só é catastrófico como é irreversível.

Outro problema complexo é a dificuldade de estimar as estimativas sobre os danos e benefícios dos rastreamentos. Os cálculos e desenhos dos estudos que mensuram isso são polêmicos, variados e implicam suposições que alteram os resultados. Por exemplo, há grande e polêmica variação nas estimativas da proporção de sobrediagnósticos no caso do rastreamento do câncer de mama (0% a 50%) ^56^. Como o conteúdo da modelo da história natural desse câncer muda radicalmente se aderimos a um lado ou outro da polêmica, existente há anos (gerando dúvidas tipicamente compatíveis com o PP), os saberes mais antigos que pouco valorizam o sobrediagnóstico persistem inercialmente alimentando o modelo.

Devido à grande heterogeneidade das neoplasias malignas e dos saberes e tecnologias envolvidos nos seus rastreamentos (quando existem), cada caso de rastreamento de câncer proposto ou já existente deve ser analisado individualmente quanto ao balanço benefícios/danos. Essa análise deve envolver vários tipos de estudos e indicadores - por exemplo, o NNS (número necessário para rastrear [*screening*]) e o NNH (número necessário para causar dano [*harm*]) ^57^ -, e ser contextualizada nas circunstâncias clínico-epidemiológicas e institucionais envolvidas.

Essas dificuldades anteriormente mencionadas são intensificadas por outras, comuns a toda a medicina baseada em evidências (MBE): a não publicação e a admissão de que sejam sigilosos os dados primários em nível de paciente em pesquisas financiadas por indústrias farmacêuticas bloqueiam a checagem de resultados por cientistas independentes. Estima-se que aproximadamente metade de todos os ensaios clínicos realizados não tenha sido publicada ^58^. A maioria dos ensaios clínicos randomizados são financiados pela indústria, que sequestra a MBE para objetivos comerciais espúrios ^59^. Há progressivo consenso no reconhecimento de que os conflitos de interesse enviesam fortemente os resultados, mas a proposta de que estudos com conflitos de interesse fossem explicitamente rebaixados em valor não foi adotada. Acreditar no que a indústria publica equivale a permitir aos políticos que contabilizem os seus votos secretamente ^60^. Isso gerou uma crise na MBE reconhecida por seus próprios defensores ^61^. Esse aspecto de pouca credibilidade e fraude na produção do saber médico, mais comum e mais grave do que se supunha, complica ainda mais os aspectos propriamente epistêmicos e já complexos das metodologias e suas limitações, com incertezas e polêmicas nas avaliações de benefícios e danos dos rastreamentos.

Adicionalmente, fatores psicológicos e/ou psicocognitivos alimentam a subvalorização dos sobrediagnósticos. Há um medo excessivo disseminado do câncer, que não acompanhou o desenvolvimento e a melhoria dos tratamentos ^62^, tendente a reforçar a expansão moral preventivista da medicina para o futuro, negligenciando irracionalmente o potencial de danos da prevenção, no caso, da P2. Isso está associado ao grande estresse e desgaste emocional dos profissionais que tratam dos cânceres ^63^ ^,^ ^64^ ^,^ ^65^, com intervenções terapêuticas altamente iatrogênicas e prognósticos comumente sombrios, e se soma ao ganho secundário das recompensas subjetivas na atividade clínica na vigência do rastreamento: esse último torna o cuidado oncológico mais leve e mais curativo, devido à “diluição” dos casos graves em muitos casos sobrediagnosticados, de ótimo prognóstico. A demanda aumentada de cuidados oncológicos gerada pelos rastreamentos (que fazem aumentar a incidência dos cânceres - estimada em 30% de aumento no caso do rastreamento mamográfico ^66^) dilui os casos graves em um conjunto emocionalmente mais confortável e recompensador de cuidar, mais leve, com maior potência curativa e menor desgaste.

Esses fatores associados entre si e à supremacia preventivista, que são provavelmente poderosos motores do paradoxo da popularidade entre os profissionais de saúde ^54^, acirram os comuns vieses psicocognitivos ^67^ ^,^ ^68^ presentes na consideração dos rastreamentos e estão associados à sua complexidade (especialmente dos sobrediagnósticos e seu caráter anômalo), tornando difícil e longínqua a construção de uma abordagem crítica e racional que reconheça a gravidade dos danos e a possível aplicação do PP à P1/P2, quando cabível.

## A naturalização da iatrogenia na prevenção

Nossa hipótese é que há uma adiantada naturalização dos danos iatrogênicos na prevenção, que se constitui em grave empecilho para a consideração do PP em situações duvidosas de P1/P2 com danos graves, fruto da supremacia preventivista, dos processos antes mencionados e de outros, alguns dos quais são adiante mencionados sem detalhamento. Por exemplo, provavelmente há uma poderosa ação social difusa, permanente e silenciosa, exercida pelos médicos especialistas dedicados às doenças crônicas de alta morbimortalidade, pró-intervenções preventivas. Esses profissionais e suas instituições são de alta respeitabilidade e legitimidade social e científica ^16^.

Fatores institucionais sistêmicos também influenciam a prática clínica e de gestão dos sistemas e serviços de saúde (*guidelines*, auditorias e sistemas de avaliação e pagamento por desempenho), dificultando uma postura crítica quanto à prevenção, ao induzirem os profissionais a fazerem mais ações preventivas, incluindo práticas duvidosas, o que se soma à prática profissional cada vez mais defensiva (decisões para proteger o profissional de eventuais litígios ^69^), em ambientes em que o excesso de intervenção não é punido, mas a omissão sim ^70^ ^,^ ^71^.

Outro aspecto cultural e simbólico de fundo pode estar centralmente envolvido na promoção e sustentação da supremacia preventivista e da tolerância excessiva aos danos iatrogênicos: a potência da face religiosa da medicina moderna, descrita por Lago ^72^ com base na articulação de ideias de Foucault, Ivan Illich, Walter Benjamin e Agamben. Das três grandes forças de socialização nos países ocidentais - ciência, capitalismo e cristianismo -, a ciência agora tem vantagem por sua aliança com a tecnologia, com grande penetração nas sociedades articulada ao capitalismo. A biomedicina agrega essas três forças, configurando a sociedade de modo menos dogmático e mais pragmático ao lidar com corpo. Ao ajudar a construir o secularismo (que não desmontou ou eliminou os mecanismos de poder e influência religiosa na cultura/sociedade) e afastar relativamente a religião dos cuidados à saúde-doença, a biomedicina acabou por “herdar” certas funções ou atributos religiosos, e para entender o seu papel é necessário pensá-la como uma religião, como Walter Benjamin fez com o capitalismo.

“*A medicina, como o capitalismo, não tem uma dogmática própria. Usa os conceitos fundamentais das ciências biológicas articulados no dualismo entre um princípio maligno (as doenças e os patógenos) e um princípio benigno, representado pela cura e mediado pelas estratégias das terapias médicas* [curativas e preventivas]*. É importante observar que o princípio benigno não é a saúde, mas a própria prática cuja ação se sobrepõe à saúde. A medicalização teria atingido um nível tão avançado e pervasivo ao ponto de não ser mais um meio para o reestabelecimento da saúde* (...)*. Por isso, para Agamben, a prática médica pode ser vista como uma prática cultual, na qual a própria prática se torna o fim, ficando na sombra sua medialidade. A prática cultual da medicina, isto é, o conjunto dos procedimentos voltados para a cura* [e a prevenção]*, está assumindo uma abrangência global.* (...) *já é uma prática permanente e distribuída uniformemente ao longo da vida, como eram os rituais na vida monástica medieval. A vida inteira das pessoas se tornou uma oportunidade para o exercício da prática cultual médica, não apenas nos momentos críticos de infortúnio ou nas épocas de transição* [faz-se o tempo todo nas ações preventivas comportamentais e periodicamente nas demais]*. Essa prática cultual não é apenas recomendada e sim obrigatória, graças ao predomínio da nova religião sobre as duas competidoras, o capitalismo e o cristianismo*” ^72^ (p. 100-1).

Essa leitura sugere que com a modernidade e o secularismo uma autoridade moral-religiosa “passou” para a biomedicina, facilitando o moralismo preventivista acrítico. A grande confiança que a modernidade demanda das pessoas nos sistemas abstratos especialistas tornou-se possível, facilitada, aceitável e superlativa no caso das ações preventivas biomédicas, ao ser recheada e temperada com esse componente religioso, o qual é facilitado pela superposição objetual: a biomedicina lida com a morte, o sofrimento etc., esferas em que a religião tinha grande protagonismo. Tal caráter ritualístico-litúrgico dificulta e afasta o pragmatismo racional e crítico da prevenção.

Isso tudo parece ter gerado um “estado de opinião” generalizado favorável irrestritamente ou pelo menos excessivamente à prevenção, convergente com o valor moral positivo a ela atribuída (a coisa “certa” a fazer), sobretudo em relação às doenças crônicas e graves. A medicina de vigilância e o entusiasmo tecnológico produzem otimismo pró-intervencionista e moralista, que dificulta a crítica à iatrogenia da prevenção. A tolerância aos danos iatrogênicos de P1/P2 parece ter sido expandida muito além do que qualquer bom senso e pragmatismo informados eticamente poderiam julgar razoável, tornando-se naturalizada em grande escala, produzindo um “novo normal” já parcialmente em vigor.

Configura-se uma situação meio surreal, como se vivêssemos em uma bolha avassaladora, supostamente científica, englobando a sociedade inteira, de otimismo ingênuo tecnológico preventivista, inflada e alimentada por interesses econômicos, propaganda viciada, manipulação e ocultação de evidências científicas e moralismo preventivista quase ou parcialmente religioso, que deveriam ser objetos de pesquisa e debate científicos depurados de interesses econômicos, mas que não podem ser depurados de contextos ideológicos, valores morais e do imaginário social.

Pode parecer que essa discussão se restringe, no Brasil, ao aproximadamente 1/4 da população que é usuária de planos de saúde ^73^ e por isto têm mais acesso ao cuidado profissional e a ações preventivas, ou a locais com APS mais bem organizada e acessível. Todavia, na falta de estudos abrangentes sobre o tema, a vivência cotidiana em diversificados serviços de APS indica que também no grosso da população brasileira urbana em pequenas e grandes cidades (mais de 87% dos brasileiros ^74^), e em grande parte das áreas rurais, há também certa supremacia preventivista ou pelo menos um disseminado e claro valor moral genérico sobre a prevenção como o “certo a fazer” - ainda que restrito a um “superego” pouco praticado, abafado, apenas parcialmente existente ou incipiente (por ser inviável ou difícil devido à falta de acesso ao SUS e/ou a adversidades e vulnerabilidades socioeconômicas intensas), explorado emocionalmente por campanhas e pelo mercado, dado não haver nenhum rastreamento organizado implantado no Brasil - apesar de alguns meritórios (mas insuficientes) esforços governamentais para mudar isso e da “quase organização” do rastreamento do câncer de colo uterino.

Nesse panorama sociocultural de ampla hegemonia ideológica preventivista, parece haver um forte alinhamento a orientar o processamento e a avaliação dos resultados das pesquisas para a supervalorização de qualquer possibilidade marginal de benefício de uma ação preventiva como uma maravilha tecnológica, clínica e sanitária, necessária e moralmente desejável, quase obrigatória. Isso alimenta a tolerância excessiva à iatrogenia da prevenção e torna aparentemente racional e moralmente aceitável a manutenção ou introdução de novas medidas preventivas de P1/P2 mesmo que duvidosas, com pequenos benefícios e danos potenciais graves. Uma vez estabelecida a presença rotineira e mantida dessas ações e respectivos danos, eles passam a angariar não mais que indiferença e resignação, que desaguam em sua aceitação geral, convertendo esses danos, com o passar do tempo, em incontornáveis e inescapáveis, normais e naturais. O corolário (científico, social, institucional e clínico) dessa naturalização é o afastamento sem debate da possibilidade de invocação e aplicação do PP em ações preventivas produtoras de danos graves ^15^ ^,^ ^17^ ^,^ ^18^.

## Considerações finais

Apesar dos requisitos consensuais para a aplicação do PP estarem presentes em algumas ações preventivas gravemente danosas, diversos fatores associados e sinérgicos fomentam a ausência de aplicação/discussão do PP, dentre os quais destacamos: a semelhança moral e teleológica entre o PP e a prevenção e suas diferenças contextuais; a ampla, forte e crescente expansão de uma supremacia preventivista, indutora de otimismo e intervencionismo preventivos e excessiva tolerância aos danos iatrogênicos; as complexidades epistêmicas da avaliação/reconhecimento dos sobrediagnósticos; o traço religioso da biomedicina na modernidade tardia, indutor de forte moralismo preventivista; a naturalização hoje avançada das iatrogenias preventivas na medicina, na saúde pública e na sociedade/cultura em geral.

A consideração desses fatores facilita a compreensão da ausência do uso do PP quando cabível na avaliação de ações preventivas, e indica a necessidade de pesquisa, discussão e ação sobre o tema. Tal necessidade pode ser traduzida como produção e disseminação de um ceticismo dirigido às ações preventivas, intensificado devido à grande valorização consensual da não-maleficência em P1/P2. Isso pode ser caracterizado como a parte preventiva do que Stegenga ^75^ chamou de “niilismo médico”. Para o autor, há suficientes evidências para um rebaixamento de expectativas e um olhar muito crítico para com as novas tecnologias médicas: o aprimoramento metodológico vem mostrando que, geralmente, quanto melhor a qualidade das evidências produzidas e disponíveis, há tendência de menores benefícios e maiores danos iatrogênicos.

O niilismo médico de Stegenga ^75^ necessita ser ainda maior nas ações preventivas aditivas (intervenções artificiais) e especialmente nos rastreamentos. O PP pode e deve ser um recurso desse ceticismo preventivo. Sua tematização em casos específicos (por exemplo, nos rastreamentos de câncer de próstata e de mama, ambos bem estudados) deveria ser discutida na comunidade científica e sanitária, na formação médica, na construção de recomendações preventivas pelo SUS e outras instituições, na sociedade, na mídia e em campanhas (por exemplo, dos meses azul ^76^ ^,^ ^77^ e rosa ^78^ ^,^ ^79^).

## Data Availability

Não se aplica.
